# Unamplified, Long-Read Metagenomic Sequencing Approach to Close Endosymbiont Genomes of Low-Biomass Insect Populations

**DOI:** 10.3390/microorganisms10030513

**Published:** 2022-02-26

**Authors:** Joseph R. Petrone, Alam Muñoz-Beristain, Paula Rios Glusberger, Jordan T. Russell, Eric W. Triplett

**Affiliations:** Microbiology and Cell Science Department, Institute of Food and Agricultural Sciences, University of Florida, Gainesville, FL 32603, USA; josephpetrone@ufl.edu (J.R.P.); amunozberistain@ufl.edu (A.M.-B.); priosglus@ufl.edu (P.R.G.); russell.j.7@ufl.edu (J.T.R.)

**Keywords:** psyllid, insect metagenome, next-generation sequencing, Oxford Nanopore, PacBio, low-biomass, unamplified, genomics, long-read assembly

## Abstract

With the current advancements in DNA sequencing technology, the limiting factor in long-read metagenomic assemblies is now the quantity and quality of input DNA. Although these requirements can be met through the use of axenic bacterial cultures or large amounts of biological material, insect systems that contain unculturable bacteria or that contain a low amount of available DNA cannot fully utilize the benefits of third-generation sequencing. The citrus greening disease insect vector *Diaphorina citri* is an example that exhibits both of these limitations. Although endosymbiont genomes have mostly been closed after the short-read sequencing of amplified template DNA, creating de novo long-read genomes from the unamplified DNA of an insect population may benefit communities using bioinformatics to study insect pathosystems. Here all four genomes of the infected *D. citri* microbiome were sequenced to closure using unamplified template DNA and two long-read sequencing technologies. Avoiding amplification bias and using long reads to assemble the bacterial genomes allowed for the circularization of the *Wolbachia* endosymbiont of *Diaphorina citri* for the first time and paralleled the annotation context of all four reference genomes without utilizing a traditional hybrid assembly. The strategies detailed here are suitable for the sequencing of other insect systems for which the input DNA, time, and cost are an issue.

## 1. Introduction

Studying the endosymbiont populations of non-model insects can be challenging due to the lack of ideal conditions and available information on organism-specific extraction optimizations. These setbacks can appear due to a lack of sample availability, DNA extraction yield and quality, or an irregularly dispersed bacterial species of interest among an infected insect population [1]. Although DNA extraction and sequencing can still be accomplished under these constraints, the ultimate goal is to achieve this with the use of cost-effective and readily available techniques [2].

In cases where the DNA yield from a single insect is below the requirement for next-generation sequencing (NGS), such as mosquitos, whiteflies, aphids, ticks, and psyllids [1,3,4,5,6,7,8,9], whole genome amplification (WGA) may be required to obtain enough DNA for downstream analysis [10]. This amplification process can introduce bias through inherent polymerase error rates, exclusion biases of smaller and low-abundance fragments [11], and the amplification of contamination in metagenomic sequencing [12]. Additionally, PCR-based WGA methods can lead to uneven genome coverage and incorrect assemblies [13], and these risks increase with AT-rich genomes [14].

We chose to employ techniques to sequence the complete endosymbiont system of *Diaphorina citri* to improve upon the quality and completeness of genomic assemblies. This small insect represents a case where it is common to observe low-DNA yields after traditional extraction techniques [4,5,6,7]. Three obligate bacterial symbionts live within the psyllid insect vector: *Candidatus* Profftella armatura, *Candidatus* Carsonella ruddii, and the *Wolbachia* endosymbiont of *Diaphorina citri. Candidatus* Liberibacter asiaticus, the causative agent of Huanglongbing (HLB) or citrus greening disease, can also infect *D. citri* populations with an uneven distribution and titer [15]. At the time of the generation of this manuscript, all the closed genomes deposited for the endosymbionts of *Diaphorina citri*, including *Ca.* L. asiaticus, first required an increase in the amount of genomic DNA quantity via amplification-based methods such as multiple displacement amplification (MDA) [16,17], the REPLI-g minikit [5,18], GenomiPhi [6,7], and primer-walking [19] before the use of short-read sequencing technologies. As for the *Wolbachia* endosymbiont, the only available assembly was incomplete [20]. Two of the endosymbionts in this system, *Ca*. P. armatura and *Ca.* C. ruddii, also increased the chance that AT-rich amplification bias affected their assemblies, with respective AT contents of 75.8% and 82.2%.

Here, the entire endosymbiont metagenomes of highly infected *Ca*. L. asiaticus-positive psyllids were assembled and closed using unamplified template DNA extracted using readily available techniques and sequenced using two third-generation, long-read technologies. The resulting assemblies and annotations were compared to currently deposited reference strains to assess if unamplified, long-read sequencing would improve upon the current depositions. The analysis of these new genome sequences provides more accurate metabolic modeling of the *Wolbachia* strain of *Diaphorina citri* and strengthens the confidence in the use of the consensus sequence for the remaining three endosymbiont genomes.

## 2. Materials and Methods

### 2.1. Insect Colony and DNA Extraction

An infected *Diaphorina citri* colony was continuously maintained at the Citrus Research and Education Center, University of Florida (CREC-IFAS, UF, Lake Alfred, FL, USA). Psyllids were reared on *Citrus macrophylla* and *Citrus sinensis* in a USDA-APHIS/CDC-approved growth room. Over 250 psyllids were individually subjected to extraction using a lab-modified E.Z.N.A. tissue kit protocol (Omega Bio-tek, Norcross, GA, USA). Washed psyllids were individually extracted using a silica-column DNA extraction kit modified to prevent further DNA fragmentation. Wide-bore pipettes and the exclusion of aggressive vortexing steps were also utilized for this purpose. Samples were aseptically crushed in 200 µL TL buffer in a V-bottom, 1.5 mL microcentrifuge tube using a sterile pipette tip bent in the shape of a “U”. This was done to lightly break up the insect without the use of the mechanical grinding of pestles or bead-beating. DNA was eluted in 100 µL of sterile nuclease-free water after heating the silica column and water for 5 min at 65 °C.

### 2.2. Phenol-Chloroform Extraction

To improve the length of DNA recovered from psyllids to close the *Wolbachia* genome, phenol-chloroform extraction was performed. Twenty additional psyllids from the same population were lightly homogenized in a microcentrifuge tube with lysis buffer for 2 h at 60 °C in a water bath and then subjected to a phenol-chloroform extraction as described previously [21]. The resulting DNA was unable to be removed via a “hook” and so was instead centrifuged into pellets, washed twice in 70% EtOH, and rehydrated in 100 µL of elution buffer.

### 2.3. Quantitative PCR

Primers were previously designed for qPCR applications to produce a small 131 bp amplicon from a gene present at a single copy within the *Ca.* L. asiaticus genome, *terC*. This primer set was also tested for specificity to *Ca.* L. asiaticus (Figure S1). The primer sequence created was (F: 5′CACCGAGATTGTATGGCTTGA3′) (R: 5′GAGCGGACACTATCCCAATAA3′). For a standard PCR 30-cycle, to verify the presence/absence of specificity, the primer set was tested on DNA from *Ca.* L. asiaticus (+/−) citrus midribs, *Ca.* L. asiaticus (+/−) psyllids, *Liberibacter crescens, Bacteroides dorei, Rhizobium meliloti,* soil from a citrus grove, and a water and algal sample from Lake Alice at the University of Florida campus (Figure S1). The amplification conditions of the primers are as follows: initial denaturation at 95 °C for 5 min, followed by 30 cycles of 95 °C for 30 s, 61.4 °C for 30 s, 72 °C for 30 s, and a final elongation step of 72 °C for 5 min.

For the quantification of the *Ca.* L. asiaticus titer in each psyllid extraction, the *terC* primers were used for qPCR. To generate the standard curve, purified *terC* amplicons from traditional PCR were quantified using Qubit 2.0 fluorometer and HS 1× buffer (Thermo Fisher Scientific, Waltham, MA, USA). The copy number of the standard curve was calculated based on the amplicon size and qubit concentration. The amplicon size was predicted to be 131 bp based on primer binding specificity in the *Ca.* L. asiaticus genome and confirmed via comparison to a 100 bp ladder in DNA electrophoresis. One microliter of DNA from individual psyllid extractions was then subjected to qPCR (QuantStudio 3, Applied Biosystems) in technical triplicates to quantify the *Ca.* L. asiaticus titer relative to the standard curve. The collection of individual psyllid extractions was then analyzed based on the copy number of *Ca.* L. asiaticus.

### 2.4. DNA Sequencing

Sequencing on the Nanopore GridION Mk1 was performed using a modified rapid sequencing kit (SQK-RAD0004) with 0.5× of tagmentation enzyme (FRA) and run on an R9.4 flow cell. Base-calling of the results from the 48-h run was accomplished using guppy v3.2.1 fast_941. The remaining DNA was sent to the University of Florida Interdisciplinary Center for Biotechnology Research (UF ICBR) to be sequenced on one lane of a SMRT cell using the PacBio Sequel platform. TapeStation fragment size analysis was performed on the end-prepped and purified PacBio portion before loading. Consensus reads were generated from the run using SMRT Link v7.0.0.

Secondary Nanopore sequencing was performed on the GridION platform using phenol-chloroform extraction. This second sequencing was conducted using the ligation sequencing kit (SQK-LSK109) and bead-free ligation prep [22] on an R10.3 flow cell with 2.1 µg of input DNA. After 48 h, the resulting fast5 files were basecalled using guppy v3.2.8 with high-accuracy basecalling mode.

### 2.5. Assembly and Polishing

Multiple assembly and polishing software processes were run on the contigs (Table 1) and the resulting consensus was annotated using RASTtk v2.0 for preliminary annotation without backfilling gaps or frameshift correction [23]. The multiple annotated genomes were then aligned to the GenBank files of currently available NCBI depositions using Mauve v2.4.0 to assess the LCB alignment of the scaffolds before submission [24]. The chosen assembly and polishing pipeline varied across genomes but was determined by manually finding the assembly conditions that resulted in a circularized contig of each bacterial species of interest. The recommended polishing pipeline based on the assembler software used was then performed for each assembly. All assembler versions and software used in assembly and polishing are listed in Table 1. Data were stored and analyzed via bioinformatic software modules installed on the University of Florida HiPerGator Research Computing cluster.

### 2.6. Genome Comparison

For nucleotide-level similarity to the genomes, the JSpeciesWS v3.9.0 webserver was used for whole-genome alignment [25]. Polished genomes from this publication were uploaded, along with all deposited and closed NCBI genomes for each endosymbiont. The average nucleotide identity of the genomes was analyzed in a pairwise fashion using MUMmer v3.0 as a part of JSpeciesWS [26]. The Circoletto v07.09.16 webserver was then used to visualize whole-genome alignments of this publication’s genomes as the reference against the most similar genome depositions, as found using JSpecieWS [27]. Absolute score ribbon coloring was selected and nucleotide identity thresholds were set at (red > 99.9999%, orange ≤ 99.9999%, green ≤ 95.9999%, and blue ≤ 85.9999%).

Using a proteome comparison tool as part of the PATRIC web server [28] and RASTtk v2.0 [23], the predicted protein profiles of this publication’s genomes were individually aligned and compared to all deposited and closed NCBI genomes for each respective species on NCBI. The resulting proteomes were outputted into a Circos plot, which graphically aligned all the predicted proteins of the genomes in the comparison. The percentage identities of the individual proteins of the genomes closed in this publication were color-coded against the closest related deposited strains.

### 2.7. Peak-to-Trough Analysis

To generate depth-of-coverage plots for the genomes, the PacBio data were mapped to the polished contigs using Minimap2 v2.16 [29] and the resulting sam file was visualized using the Samtools v1.12 depth function [30], with the resulting file plotted in RStudio v1.4.1717 [31] using the ggplot package.

## 3. Results

### 3.1. Infected Psyllid DNA Pool

To generate enough endosymbiont gDNA for long-read sequencing to capture the four genomes, insects first needed to be selectively pooled from individual insect extractions of those that were infected with the highest titer of the limited bacterium, *Ca*. L. asiaticus, relative to the population. The 26 psyllids that were selected to make up the pool were confirmed via qPCR to have the highest relative titers of *Ca*. L. asiaticus (>3000 copies/µL) among the population of 250 extractions (Figure S2A). The pool consisted of an average of 2.86 × 10^4^ copies/µL and a median of 2.16 × 10^4^ copies/µL of *Ca.* L. asiaticus DNA. This method yielded a collective 1.2 µg of DNA, which were then split into two tubes. Four hundred nanograms were used for in-lab Oxford Nanopore sequencing (ONT) on the GridION Mk1 platform (Oxford, UK), whereas the remaining portion was sent to the ICBR to be run on the Pacific Biosciences (PacBio) Sequel platform. After the final AMPure cleanup step during ICBR PacBio library preparation, a primary peak of 1.15 ng/µL at 11.7 kb was observed in the psyllid DNA pool with fragments ranging from 1 kb to 30 kb (Figure S2C).

### 3.2. Initial Sequencing Results and Genome Assembly

The initial Nanopore sequencing portion was conducted using a 400 ng aliquot from the pooled 1.2 µg of gDNA from *Ca*. L. asiaticus positive psyllids. The run resulted in 3.55 × 10^5^ reads with an average length of 920 bases and a total output of 286 Mb. As for the other long-read technology, the prepared PacBio library was run on a single SMRT cell lane for 1200 min, resulting in 34.8 Gb and 1.33 × 10^7^ subreads, with a median subread length of 3.11 Kb. All fastq files from the runs have been submitted to the Sequencing Reach Archive database under accession number SRS5945824.

*Ca.* Liberibacter asiaticus JRPAMB1 was assembled from the PacBio sequel data alone using HGAP4 (Table 1) as part of SMRT Analysis Software v7.0.0 with the genome size set to 4.5 Mb [32]. The assembled contig had an average coverage of 1283×. “JRPAMB” was given as the numbered strain names for the genome depositions to represent the initials of the first two authors. *Ca*. Profftella armatura JRPAMB3 and *Ca*. Carsonella ruddii JRPAMB4 were assembled using Canu v1.8 [33] with the initial Nanopore sequencing data with the anticipated genome size set to 500 Kb and the anticipated error rate set to 0.85. The assembly coverage of the *Ca*. Profftella armatura and *Ca*. Carsonella ruddii genomes were initially 24.2× and 16.03×, respectively. All assemblies for *Ca*. C. ruddii yielded slightly missing ends of the contigs relative to *Ca*. C. ruddii str. DC. The missing portion of this genome was closed after re-shuffling the start position of the fasta file and subjecting the contig to a round of Circulator v1.5.5 processing [34] with all the Nanopore and PacBio data combined. This area now corresponds to a stretch of 2500 bp within the rrn operon of *Ca*. C. ruddii.

The genome of *Ca*. L. asiaticus JRPAMB1 was polished by running an additional round of Arrow via SMRT Link v7.0.0. *Ca*. P. armatura and *Ca*. C. ruddii were both assembled using the initial Nanopore sequence data but benefited from polishing using PacBio reads as well. The *Wolbachia* and *Ca*. P. armatura contigs benefited from four rounds of polishing using the Minimap2 v2.16 [29] and Racon v1.4.3 [35] polishing combination with the PacBio reads, whereas *Ca*. C. ruddii benefited from two rounds of the SMRT Analysis v7.0.0 resequencing workflow using the Arrow polisher.

### 3.3. Long-Read Sequencing Leads to the First Circularization of the Wolbachia Strain Genome

Although the *Wolbachia* isolate was assembled through our initial efforts and was reported as circularized after our first HGAP4 assembly, which resulted in the genome of *Ca.* L. asiaticus JRPAMB1 using the PacBio data, an additional contig of approximately 273 Kb was noticed in the assembler output files. This smaller contig was missing from the main “circularized” assembly but was present in *Wolbachia* strain wACP3, along with other *Wolbachia* insect systems. This contig contained a large number of repeat-rich regions spanning transposable elements, suggesting that reads long enough to cover these regions for the assembler context were not present. Closing the genome required additional sequencing on the Oxford Nanopore GridION system using the DNA from a separate phenol-chloroform extraction to generate long enough reads to bridge over these two contigs. Furthermore, 2.1 µg of high-molecular-weight DNA was taken forward using a bead-free ligation protocol specific to Nanopore [22]. Sequencing using the updated R10.3 flow cells allowed for higher throughput and a better raw error rate. Furthermore, 6.6 Gb of basecalled data were generated, producing 1.31 × 10^6^ reads with an N50 of 17 Kb. Flye assembler v2.8.1 [36] was run with all the sequencing data from the three runs as inputs to resolve the assembly. With a complete, circularized genome of *Wolbachia* from *D. citri* now available, this strain of *Wolbachia* was given the isolate name “dawsonii” in honor of the great citrus pathologist Professor Emeritus William O. Dawson of the University of Florida.

### 3.4. Whole-Genome Nucleotide Similarity of the Genomes

**Figure 1:**
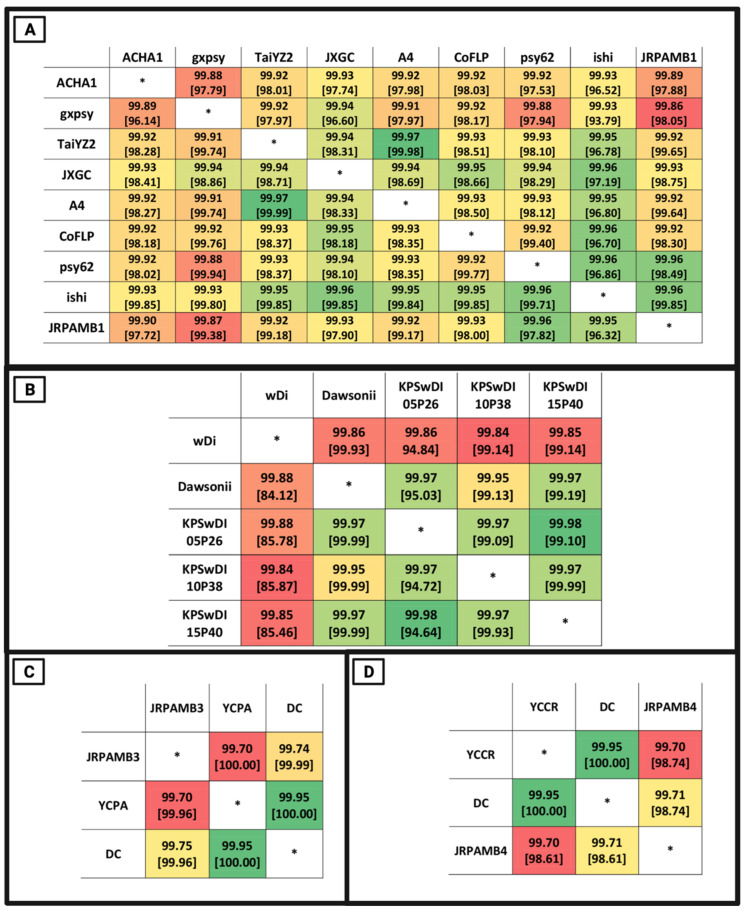
Whole-genome nucleotide identity as calculated through MUMmer v3.0. The query genome is listed in the first column, whereas the subject genome is listed under the first row. The metrics inside of the boxes reflect the nucleotide identity percentage (top) and percentage query coverage (bottom in brackets) for the query against the respective subject. The heatmap coloring scheme denotes “green” as the highest nucleotide percentage identity match and “red” as the lowest nucleotide percentage identity. The coloring is relative, meaning that a “red” color does not necessarily mean a low-quality comparison. Asterisks indicate self-alignment and are excluded. (**A**) Nucleotide identity statistics of *Ca*. L. asiaticus strains. (**B**) Nucleotide identity statistics of *Wolbachia* strains. (**C**) Nucleotide identity statistics of *Ca*. P. armatura strains. (**D**) Nucleotide identity statistics of *Ca*. C. ruddii strains.

**Figure 2:**
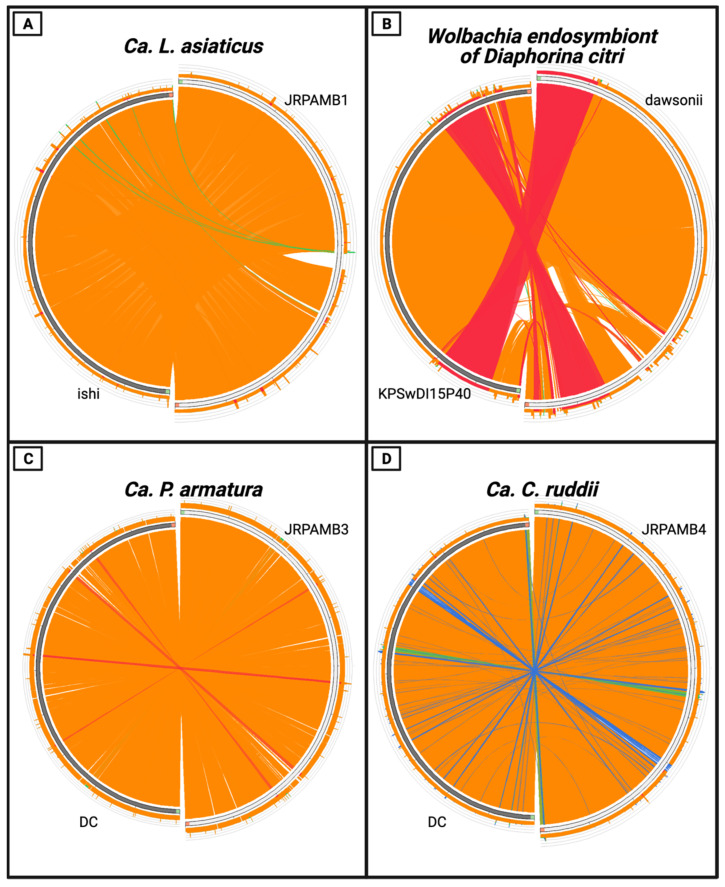
Circoletto plot comparing the nucleotide LCBs of this publication’s strains (right) to the closest relative strains (left) based on the MUMmer ANI across the genome position. The strain on the right of each Circoletto plot was used as the query for the search. An E-value cutoff of 1 × 10^−40^ was used for each construction. The size of each ribbon represents the size of the alignment, whereas the color corresponds to the percent identity of the alignment (red > 99.9999%, orange ≤ 99.9999%, green ≤ 95.9999%, blue ≤ 85.9999%). The absence of a ribbon indicates an area unique to that genome under the search constraints. (**A**) *Ca*. L. asiaticus str. JRPAMB1 (right) against the nucleotides of str. ishi. (**B**) *Wolbachia* endosymbiont of *D. citri* str. dawsonii (right) against the str. KPSwDI15P40. (**C**) *Ca*. P. armatura str. JRPAMB3 (right) against str. DC. (**D**) *Ca*. C. ruddii str. JRPAMB4 against str. DC.

The *Candidatus* Liberibacter asiaticus strain JRPAMB1 genome represents the second largest of all the closed NCBI genomes for this species at 1.237 Mb. NCBI PGAP v5.1 annotation pipeline [37] predicted 1111 genes encoding for 1032 proteins (Table 1) and most closely aligned to the ishi strain (Figure 1A) with a nucleotide percent identity of 99.95%, aligning 99.32% of the total genome of JRPAMB1 to the ishi strain (Figure 2A).

The *Wolbachia* endosymbiont of *Diaphorina citri* isolate dawsonii was the first complete genome for this endosymbiont at the time of submission. Annotation of the 1.66 Mb genome predicted 1561 genes coding for 1332 proteins (Table 1). This *Wolbachia* isolate dawsonii genome covered all 124 contigs of the wACP3 *Wolbachia* genome [20], which was previously mined from the data of the *D. citri* genome cohort [38] (Figure 1B). Additionally, during the publication of this article, three genomes of a *Wolbachia* strain from *Diaphorina citri* passaged in *Drosophila* S2 cells were deposited but presumed to be incomplete [39]. The genome of the *Wolbachia* isolate dawsonii entirely covered the recent submission of three versions and most closely aligned with isolate KPSwDI05P40, with a whole-genome nucleotide identity of 99.97%, aligning 99.19% of isolate dawsonii nucleotides (Figure 2B).

*Candidatus* Profftella armatura strain JRPAMB3 represents a closed genome for the species and has a length of 461,109 bp with a 5459 bp plasmid. *Ca*. P. armatura JRPAMB3 has a GC content of 24.2% (Table 1) and is most closely related to *Ca*. Profftella armatura DC (Figure 2C) with 99.74% nucleotide identity (Figure 1C). *Candidatus* Carsonella ruddii strain JRPAMB4 has a closed assembled length of 174,118 bp and a GC content of 17.8% (Table 1). Our strain shares 99.71% nucleotide identity, aligning 98.61% of the genome to Carsonella ruddii str. DC (Figure 1D and Figure 2D).

Both *Ca*. L. asiaticus JRPAMB1 and *Wolbachia* dawsonii contain the gene encoding for DnaA helicase in their genomes. The genome start positions have been set to reflect the *dnaA* gene on the positive strand and the start codon as nucleotide position one in the genomes. For *Ca*. P. armatura and *Ca*. C. ruddii, the absence of the *dnaA* gene prevented the genomes from being traditionally orientated. Instead, we chose to position the genome to mirror the start positioning and the strand of their closest deposited strain. For *Ca*. P armatura and *Ca*. C. ruddii, the starting positions were set to pmbA and tmrE, respectively.

All the genomes deposited from this publication were documented as closed, circularized, and complete. CheckM v1.1.2 analysis of these genomes suggests that compared to their NCBI closest depositions, these genomes were identical in completeness and contamination (Table 1) [40]. Discrepancies in the “completeness” level of the genomes listed in Table 1 can be explained due to the low CheckM completeness levels found in small bacterial genomes, despite being otherwise complete [41,42].

### 3.5. Protein-Level Comparison of the Genomes

**Figure 3:**
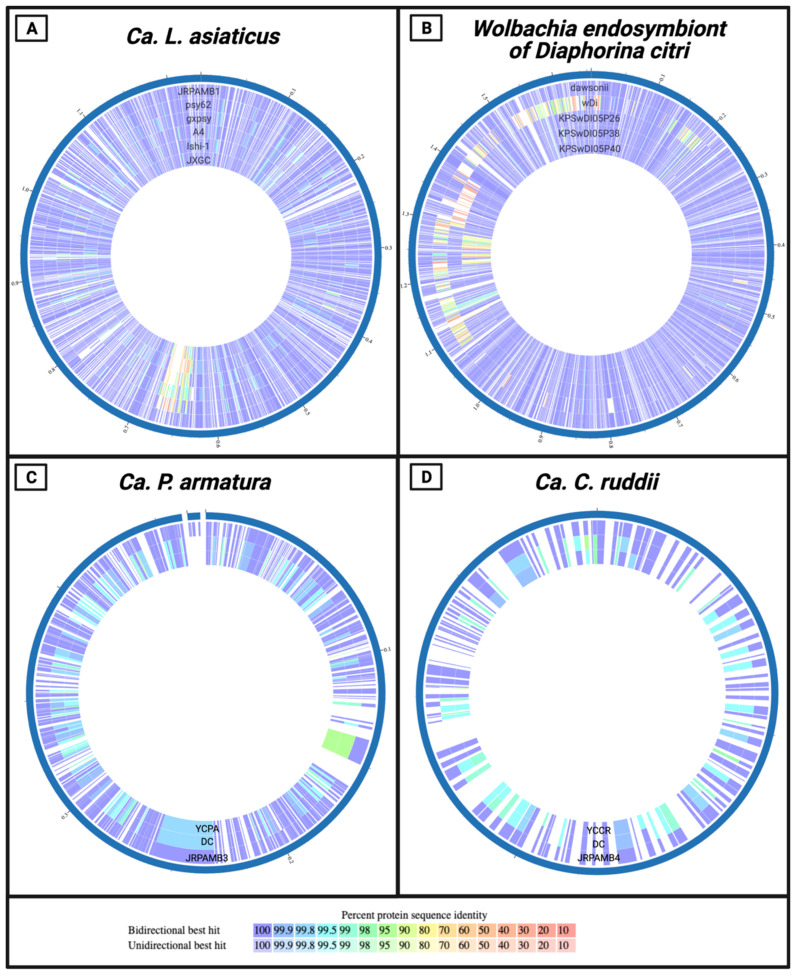
Circos plots comparing the predicted proteomes of our JRPAMB strains 1–4 on the outermost ring to the current NCBI depositions. (**A**) The genome of *Ca*. Liberibacter asiaticus JRPAMB1 against (outer to inner) str. psy62, str. gxpsy, str. A4, str. Ishi-1, and str. JXGC, respectively. (**B**) *Wolbachia* dawsonii contigs against wACP3, KPSwDI05P26, KPSwDI05P38, and KPSwDI05P40. (**C**) *Ca*. Profftella armatura JRPAMB3 against NCBI strains DC and YCPA. (**D**) *Ca*. Carsonella ruddii strain JRPAMB4 vs. strains DC and YCCR. Created with BioRender.com.

At the protein level, the newly assembled genomes nearly mirrored the consensuses of their closest deposited strains. Comparing the proteomes holistically, almost all the predicted proteins and the corresponding amino acid sequences of *Ca*. L. asiaticus JRPAMB1 and *Wolbachia* isolate dawsonii aligned with a percentage identity of 100% to their closest NCBI deposition (Figure 3A,B). With the exception of the variable prophage regions of *Ca*. L. asiaticus (position ~650 Kb) and *Wolbachia* (position ~1.25 Mb), it can be seen that the percentage identities were still around 99% when not identical. For the genomes of *Ca*. P. armatura JRPAMB3 and *Ca*. C. ruddii JRPAMB4, both initially assembled with the first Nanopore sequence data and polished with PacBio, the protein identity level was slightly less similar than for JRPAMB1 and dawsonii to their NCBI counterparts. The majority of the proteins aligned at or above 99% identity (Figure 3C,D), but a few proteins from each genome mapped with around 95% identity (shown as green). The protein locus tags, percentage identities, and query coverage for each comparison in the Circos plot can be found in Table S1.

### 3.6. Peak-to-Trough Ratios Suggest Ca. Profftella Armatura Actively Replicating in the Population

**Figure 4:**
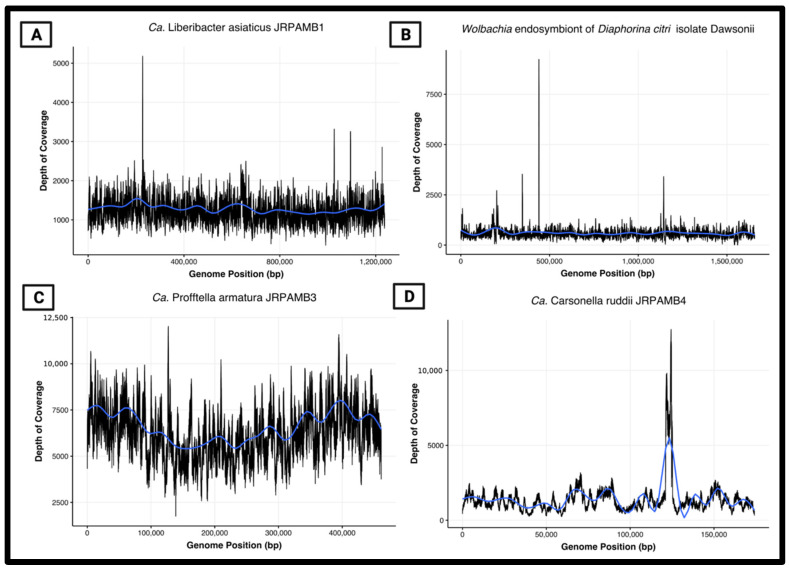
Depth of coverage vs. position of mapping to reference genome. Plots were generated in RStudio by mapping the PacBio run data to this publication’s submitted consensus genomes as reference using minimap2. The blue line represents the geom_smooth of RStudio ggplot regression averages. The x-axis refers to the nucleotide base pair position (bp) position of the genome from the start of the file. (**A**) *Ca*. L. asiaticus str. JRPAMB1. (**B**) Wolbachia str. dawsonii. (**C**) *Ca.* P. armatura str. JRPAMB3. (**D**) *Ca*. C. ruddii str. JRPAMB4.

A benefit of using unamplified template DNA for this analysis was the ability to retain accurate read coverage when mapping the raw reads to the assembled contigs. When read coverage is plotted along the length of the contig, the uneven read coverage of actively dividing cells can be visualized because genome replication is arrested at different points during extraction. The peak-to-trough ratios (PTRs) of read coverage at the origin of replication, *oriC*, compared to the termination site, *ter*, can then be used to estimate the growth rate of the population of cells used to construct the genome [43]. In our analysis of a selection of psyllids containing a high titer of *Ca*. L. asiaticus, we found the PTRs to represent mostly slow-growing populations or those that are not actively dividing at a detectable level for a population. The depth of coverage of the *Ca*. L. asiaticus, *Wolbachia* dawsonii, and *Ca*. C. ruddii assemblies appeared to be evenly mapped along the genomes with no observable differences in coverage, and *oriC*/*ter* ratios were around 1.0 (Figure 4A,B,D). In contrast, the *Ca*. P. armatura coverage plot showed a visible peak and trough in coverage depth across the genome (Figure 4C), indicating an increased copy number of reads mapping to an actively dividing *oriC*. Although *Ca*. P. armatura does not possess the DnaA helicase in its genome, the GC skew content can be used to show where the unwinding of the *oriC* takes place and where the *ter* site may be located [44,45]. Using a nucleotide skew algorithm (GenSkew, TU Munich), the predicted *oriC* and *ter* sites of *Ca*. P. armatura were determined to be around 461 bp and 262 kb, respectively [46], which corresponds to around the highest and lowest depth in the coverage plot (Figure 4C). This PTR of around 1.6 suggests that compared to the other endosymbionts, *Ca*. P. armatura may have the fastest predicted replication rate in this population [47].

## 4. Discussion

Input DNA quantity and quality, the limiting abundance of variable bacterial populations, and cost may be constraints in the whole-genome sequencing applications of insect microbiomes. We have demonstrated the ability to individually extract total DNA from low-biomass insects and utilize qPCR techniques to pool samples with the highest titer of a desired bacterial target, without the need for WGA techniques. The use of readily available silica-based DNA extraction kits still permitted the retention of long DNA fragments taken forward for long-read sequencing. The choice of using the rapid kit versus ligation for Nanopore sequencing was to prevent DNA loss during size selection and to utilize the fastest protocol to generate data. However, the adapter complex used in the rapid kit uses a transposase that randomly fragments the DNA to attach the adapter, which accounts for the vast difference in average read lengths between PacBio and Nanopore from the same template genomic DNA.

Ultimately, a pooled phenol-chloroform extraction process was needed to bridge regions with a high frequency of transposable repeats to close the *Wolbachia* endosymbiont of the *Diaphorina citri* isolate dawsonii genome for the first time. Based on surveying a population of psyllids for genetic material, it is also likely that this transposable segment was found in different genomic regions within the sample pool, leading to errors in the assembly software in calling a consensus sequence. This evidence suggests that the use of individual phenol-chloroform extractions and subsequent qPCR analysis to pool for a target bacterium may be the most effective technique to retain long enough reads to close highly-repetitive elements in genomes. Although all closed genomes here showed sufficient mapped read coverage solely using the PacBio data (Figure 4), the inability to generate all assembled contigs via the single dataset alone and the same assembler software shows the unpredictability involved in assembling, which can affect the price point of the needed sequencing platform and depth for specific aims.

The main disadvantage of long-read technologies such as PacBio and Oxford Nanopore is nucleotide insertion and deletions (indels), especially in homopolymer regions, of individual reads due to the higher error rate per read and the nature of the sequencing technologies [48]. Nevertheless, the prevalence of indels is quickly becoming resolved due to improvements over the years, leading to the achievement of over 99% base-calling accuracy thanks to continual updates to base-callers, improved pore membranes, and read error corrections, as well as PacBio’s new HiFi sequencing method [49]. Although traditional hybrid assemblies using ONT/PacBio and Illumina have been regarded as having the highest accuracy, it is still possible to assemble high-quality, highly contiguous genomes with long-read sequencing technologies alone [50]. With our use of high coverage and a mixed assembly/polishing approach, using two long-read technologies, we were able to create de novo assemblies with nucleotide accuracies over 99.5% when compared to the currently deposited reference genomes. Although these remaining indels appear to cause a select few frameshifting events, leading to a few additional pseudogenes than the current reference genomes, it should be noted that current annotation pipelines such as RASTtk [23] have the default option of fixing frameshifts and backfilling gaps caused by these sequencing errors in the downstream prediction process. This leads to very accurate annotation and protein predictions for bioinformatics applications, which can be seen in the high protein sequence identity of the strains in the proteome plots (Figure 3).

Although we hypothesized that amplification bias and errors resulting from amplifying insect template DNA to construct a usable library would affect the genome construction, we found that using unamplified DNA did not significantly change the resulting genetic content. Thus, whole genome amplification of template DNA in cases of scarce insect availability may be a suitable technique when constructing de novo assemblies. The *Wolbachia* endosymbiont of the *Diaphorina citri* genome, however, may be a case where the amplification of template DNA hindered the ability to produce a circularized contig, due to the high abundance of repeat-rich regions and transposable elements. Amplification of highly repetitive DNA is notably very difficult, as artifacts are expected to be introduced [51]. Nonetheless, an important benefit of using an unamplified template for library construction and DNA sequencing is the ability to study depth of coverage to infer cell-division status and this leaves open the potential to study epigenetics using native methylation status of template DNA.

## Figures and Tables

**Table 1:** Assembly statistics comparing the NCBI PGAP annotations of this publication to the closest strains deposited to NCBI sharing the highest nucleotide similarity. From left to right for NCBI strains: *Ca.* L. asiaticus str. ishi, *Wolbachia* KPSwDI05P40, *Ca.* P. armatura str. DC, *Ca.* C. ruddii str. DC. The size reported in parentheses for *Ca.* P. armatura reflects the length of the plasmid.

	*Ca.* L. asiaticus JRPAMB1/ ishi	*Wolbachia* Dawsonii/ KPSwDI05P40	*Ca.* P. armatura JRPAMB3/ DC	*Ca.* C. rudii JRPAMB4/ DC
Genes (total)	1111/1057	1561/1409	405/401	226/221
Protein CDSs	1032/980	1332/1234	360/356	198/196
tRNAs	44/44	34/34	34/34	22/22
Pseudo Genes	23/21	188/134	5/5	3/0
Size (bp)	1,237,165/1,190,853	1,656,288/1,538,623	461,109/459,399 (5459/5458)	174,118/174,014
GC Content (%)	36.4/36.3	34.0/33.9	24.2/24.2	17.8/17.6
Assembler	HGAP v4	Flye v2.8.1-meta	Canu v1.8	Canu v1.8
Polisher	Arrow Resequencing SMRT Linkv7.0.0	(4×) Minimap2v2.16 + Racon v1.4.3	(4×) Minimap2v2.16 + Racon v1.4.3	Arrow Resequencing SMRT Linkv7.0.0
Contigs	1/1	1/1	1/1	1/1
CheckM(%) Completeness Contamination	97.30/97.30 0.00/0.00	100/100 0.43/0.43	33.47/33.47 0.00/0.00	16.60/16.60 0.00/0.00

## Data Availability

The resulting sequencing output of both long-read sequencing technologies has been deposited in the Sequencing Read Archive (SRA) under submission number SRS5945824. The genome sequencing efforts have been collectively deposited under BioProject number PRJNA544530. The genome sequence of *Ca.* L. asiaticus JRPAMB1 has been deposited in GenBank under BioSample number SAMN11842353, accession number CP040636. The genome sequence of *Ca. Wolbachia* strain dawsonii has been deposited in GenBank under BioSample number SAMN12136689, accession number CP051608. The genome sequence of *Ca.* P. armatura JRPAMB3 has been deposited in GenBank under BioSample number SAMN12136777, accession number CP041281 for the genome, and accession number CP041282 for the plasmid. The genome sequence of *Ca.* C. ruddii JRPAMB4 has been deposited in GenBank under BioSample number SAMN12136778, accession number CP041245.

## Supplementary Materials

The following are available online at https://www.mdpi.com/article/10.3390/microorganisms10030513/s1, Figure S1: Agarose gel depicting the results of *terC Ca.* L. asiaticus-specific primer PCR specificity check. *Ca.* L. asiaticus-positive psyllids (Las +) were tested against DNA from uninfected psyllids (Las −), *Ca.* L. asiaticus-positive and negative plants (*Ca.* L. asiaticus +/−), citrus grove soil (soil), *Rhizhobium meliloti* (Rhiz), algae and water from L. Alice on the UF campus (Algae/L.Alice), *Bacteroides dorei* (B. Dor), and *Liberibacter crescens* growing in M15 (M2) and BM7 (B1). The anticipated amplicon size of 131 bp was detected in the PCR and qPCR results; Figure S2: (A) qPCR data of *Ca.* L. asiaticus titer in individual psyllids using 291 *terC* primers. The standard curve to determine the copy number was performed via purification of the amplicon, generated by subjecting individual psyllids to standard PCR. (B) TapeStation analysis from the ICBR of the PacBio library. (B3) A band of DNA around 12 kb was observable after all purification steps. (C) TapeStation analysis from the ICBR of the PacBio library. A peak of DNA around 12 Kb was observable after all purification steps. An additional larger peak of small fragments can be observed.
