# Supplementary material for: Unamplified, Long-Read Metagenomic Sequencing Approach to Close Endosymbiont Genomes of Low-Biomass Insect Populations
Source: Microorganisms. 2022 Feb 26;10(3):513. doi: 10.3390/microorganisms10030513 (PMC8948638; doi:10.3390/microorganisms10030513)
Supplement: Supplementary file 1 [file microorganisms-10-00513-s001.zip › microorganisms-1537812-supplementary/Supplementary/SUPPLEMENTARY FIGURES.pdf]

SUPPLEMENTARY FIGURES

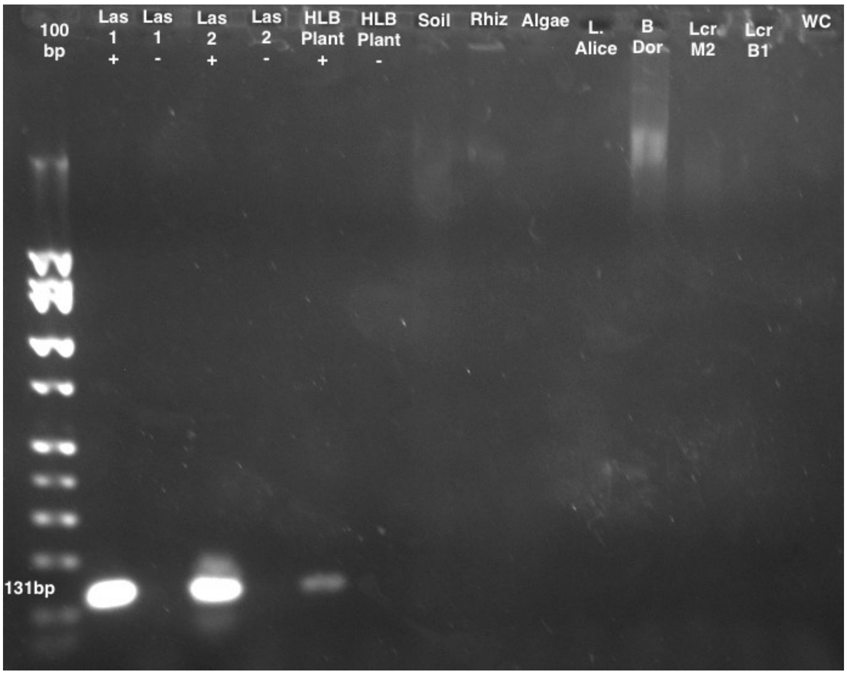

Figure S1. Agarose gel depicting the results of terC *Ca.* L. asiaticus specific primers PCR specificity check. *Ca.* L. asiaticus positive psyllids (Las +) were tested against DNA from uninfected psyllids (Las -), *Ca.* L. asiaticus positive and negative plants (*Ca.* L. asiaticus +/-), citrus grove soil (soil), *Rhizhobium meliloti* (Rhiz), Algae and water from L. Alice on UF campus (Algae/L.Alice), *Bacteroides dorei* (B. Dor), and *Liberibacter crescens* growing in M15 (M2) and BM7 (B1). The anticipated amplicon size of 131bp was detected in the PCR and qPCR results.

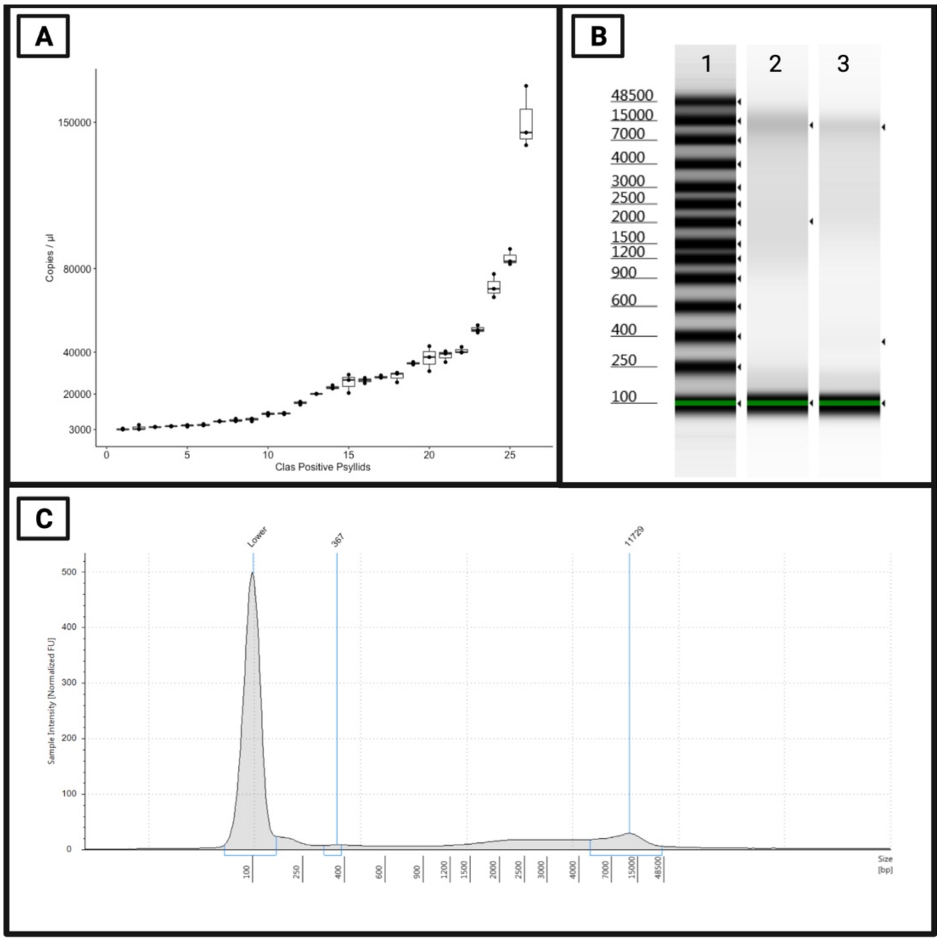

Figure S2. (**A**) qPCR data of *Ca.* L. asiaticus titer in individual psyllids using 291 TerC primers. The standard curve to determine copy number was done by purification of the amplicon generated by subjecting individual psyllids to standard PCR. (**B**) TapeStation analysis from the ICBR of the PacBio library. B3) A band of DNA around 12kb is observable after all purification steps. (**C**) TapeStation analysis from the ICBR of the PacBio library. A peak of DNA around 12 Kb is observable after all purification steps. An additional larger peak of small fragments can be observed.
